# Cytokine Profile Analysis During Sialodacryoadenitis Virus and Mouse Hepatitis Virus JHM Strain Infection in Primary Mixed Microglia and Astrocyte Culture—Preliminary Research

**DOI:** 10.3390/cells14090637

**Published:** 2025-04-25

**Authors:** Michalina Bartak, Weronika D. Krahel, Karolina Gregorczyk-Zboroch, Marcin Chodkowski, Adrian Valentin Potârniche, Ewa Długosz, Małgorzata Krzyżowska, Joanna Cymerys

**Affiliations:** 1 Division of Microbiology, Department of Preclinical Sciences, Institute of Veterinary Medicine, Warsaw University of Life Sciences, 02-786 Warsaw, Poland; weronika_krahel@sggw.edu.pl (W.D.K.);; 2 Division of Medical and Environmental Microbiology, Military Institute of Hygiene and Epidemiology, 01-063 Warsaw, Poland; 3 Department of Infectious Diseases and Preventive Medicine, Faculty of Veterinary Medicine, University of Agricultural Sciences and Veterinary Medicine Cluj-Napoca, 400372 Cluj-Napoca, Romania; 4 Division of Veterinary Epidemiology and Economics, Institute of Veterinary Medicine, Warsaw University of Life Sciences-SGGW, 02-776 Warsaw, Poland

**Keywords:** SDAV, MHV-JHM, microglia, astrocytes, ROS, apoptosis, cytokine release syndrome

## Abstract

The Coronaviridae family has again demonstrated the potential for significant neurological complications in humans during the recent pandemic. In patients, these symptoms persist throughout the infection, often lasting for months. The consequences of most of these post-infection symptoms might be linked with abnormal cytokine production and reactive oxygen species (ROS) expression, resulting in neuron damage. We investigated the effect of infection with the Mouse Hepatitis Virus (MHV) JHM strain and Sialodacryoadenitis Virus (SDAV) on a primary microglia and astrocyte culture by analysing ROS production, cytokine and chemokine expression, and cell death during one month post infection. For this purpose, confocal microscopy, flow cytometry, and a high-throughput Luminex ProcartaPlex immunopanel for 48 cytokines and chemokines were utilised. The replication of MHV-JHM and SDAV in microglia and astrocytes has increased the production of pro-inflammatory cytokines and inhibited the production of anti-inflammatory cytokines. The cytokine expression induced by the two viruses differed, as did their detection after infection. SDAV infection resulted in a much broader cytokine response compared to that of MHV-JHM. Both viruses significantly increased ROS levels and induced apoptosis in a small percentage of the cells, but without necrosis.

## 1. Introduction

The havoc that the *Coronaviridae* family has once again presented in the form of a historically catastrophic pandemic can still be observed in humans in the form of intractable and critical neurological complications. A multitude of neuropsychiatric symptoms was observed, i.e., impaired cognition, altered attention, reduced consciousness, seizures, and abnormal movements [1]. Patients experience persistent or new symptoms after a 4-week acute phase, often lasting many months [2,3,4]. These events have led to deterioration in patients with neurodegenerative diseases such as Alzheimer’s disease (AD) or Parkinson’s disease (PD) [5,6]. However, information on the connection between infection and amyotrophic lateral sclerosis (ALS), frontotemporal dementia (FTD), or Huntington’s disease (HD) is still limited [4,5,7,8,9]. The rationale for long-term COVID-19 lies in the course of infection and the immune response. The events in glial cells, particularly microglia and astrocytes, are critical to the subsequent development of neuropathy [10,11,12]. Significant cytokine output is a particular and important consequence of most COVID-19 infections. This phenomenon is referred to as cytokine release syndrome (CRS), a systemic inflammatory response triggered by infections, drugs, antibody-based immunotherapies, chemotherapeutic agents, and graft-vs.-host disease. The description of CRS provides a more balanced presentation of symptoms in patients with elevated cytokine production and clinical manifestations [13,14].

During an emergency state such as a viral infection, crosstalk between microglia cells and central nervous system (CNS) resident cells, including neurons, astrocytes, and oligodendrocytes, comes into action as a specific anti-pathogen barricade. Microglia and astrocytes are especially able to recognise invading pathogens by cellularly sensing pathogen-associated molecular patterns (PAMPs) using pattern recognition receptors (PRRs) [13,15]. In turn, they react by producing an array of inflammatory factors, including cytokines, resulting in oxidative stress, which triggers neuroinflammation [16,17]. The recognition of viruses by microglia via toll-like receptors (TLRs), RIG-I-like receptors (RLRs), and cGAS-STING pathways triggers the rapid production of interleukin 6 (IL-6), tumour nercosis factor alpha (TNF alpha), IL-1 beta, and interferon gamma (IFN gamma), which in turn activate astrocytes through direct viral detection or damage-associated molecular patterns (DAMPs) such as high mobility group box 1 protein (HMGB1) [12,18,19,20,21].

Among many cytokines, the most crucial ones in terms of viral infection are IFNs, IL-8, IL-6, IL-1, granulocyte and macrophage colony-stimulating factor (GM-CSF), TNF alpha, IL-18, IL-12, IL-2, and IL-23. Furthermore, these cytokines are involved in the induction of an immune response-type Th1/TCL with the purpose of eliminating infected cells and extracellular viruses, while cytokines such as IL-4, IL-10, IL-13, IL-37, and transforming growth factor beta (TGF beta) modulate the immune response to a Th2 and Th17 phenotype, which produce immunomodulatory and anti-inflammatory actions [22]. Moreover, in the infection course, various cytokines are produced by innate and adaptive cells that can be infected or activated. In filovirus infection, IL-1 beta, IL-5, IL-8, and IL-18, as well as various chemokines like macrophage inflammatory protein-1 alpha (MIP-1 alpha) and MIP-1 beta, monocyte chemoattractant protein 1 (MCP-1), and IFN gamma-inducible protein 10 (IP10), among others, are produced [23]. In influenza virus infection, TNF alpha, IL-1 alpha and beta, and IL-6 and IL-8 are produced [24,25,26], and hepatitis C virus can promote the expression of IL-6, IL-8, MIP-1 alpha, MIP-1 beta, and IL-1 [27], while rotavirus can induce the production of IFN, IL-8, IL-6, IL-1, TNF alpha, IL-18, IL-12, IL-2, and IL-23 [28,29,30]. Murine Hepatitis Virus strain A59 (MHV-A59) brain infection elevates five proinflammatory cytokines: TNF alpha, IL-1 beta, IL-6, IL-12p40, and IL-15 [31,32]. The cytokine signature differs when coronavirus infects the brain compared to when the virus stays outside the central nervous system [33].

The glia consists of microglia (immune cells), oligodendrocytes and astrocytes (of neural origin). These cells differ in their activity and the role they play in the homeostasis of CNS [34]. Microglia are the resident mononuclear phagocytes of the CNS that originated from yolk-sack, accounting for 5–12% of cells in the adult mouse brain [35] and 0.5–16.6% of cells in the adult human brain [36]. The activation of microglia in the CNS is heterogenous, with two opposite phenotypes—pro-inflammatory M1 and anti-inflammatory M2 [37]. Polarisation towards the M1 phenotype occurs upon exposure to brain-invading pathogens, cellular debris, and pro-inflammatory cytokines (IFN gamma and TNF alpha). This leads to the production of pro-inflammatory cytokines (TNF alpha, IL-1 beta, IL-6, and IL-2), chemokines, nitric oxide (NO), reactive oxygen species (ROS), reactive nitrogen species (RNS), and superoxide. The induction of M2 phenotype is caused by anti-inflammatory cytokines (IL-4 and IL-13), leading to the production of anti-inflammatory IL-10, TGF beta, insulin-like growth factor 1 (IGF-1), fibroblast growth factor (FGF), and colony-stimulating factor 1 (CSF-1) [38,39,40,41,42]. The M1 phenotype can be switched to the M2 phenotype by many factors [43]. Similarly to microglia, astrocytes, supporting cells of the CNS, can also be polarised into a pro-inflammatory, neurotoxic A1 type and neuroprotective A2 type [44,45]. Crosstalk between astrocytes, microglia, and neurons, based on neuro-immune communication, is fundamental to brain homeostasis [46,47,48].

In the present study, we investigated the effect of two animal batacoronaviruses—Mouse Hepatitis Virus (MHV) and rat Sialodacryoadenitis Virus (SDAV) on a mixed primary culture of microglia and astrocytes in vitro, with a particular focus on analysing the expression levels of cytokines secreted in response to infection. MHV strain JHM (MHV-JHM) is neuropathogenic, and SDAV possesses high zoonotic and neurotropic potential. To date, SDAV research has not considered the importance of CNS infections. Few publications confirmed the possibility of brain infection by SDAV [49], but no molecular pathway has been researched yet. MHV-JHM, on the other hand, is a well-known reference model for neuropathogenic diseases. The demyelination observed in MHV-JHM infection is also considered immune-mediated. While microglia are crucial for initiating the immune response, their continued activation can lead to excessive inflammation that damages not only virus-infected cells but also uninfected oligodendrocytes and neuronal cells [32,50,51,52,53].

## 2. Materials and Methods

### 2.1. Primary Mixed Microglia and Astrocyte Culture

Mixed microglia and astrocyte cell cultures were obtained from the whole brains of neonatal BALB/c mice. The blood vessels and meninges were carefully removed from the brain. Then, the whole mice brains were pooled together, washed with cold phosphate-buffered saline (PBS), and digested with a 2.5% trypsin EDTA-free solution for 15 min at 37 °C. Again, after incubation, the cells were washed five times in a warm PBS solution and mechanically homogenised using a pipette. Subsequently, the homogenate was filtered through a 70 μM cell strainer (BD Biosciences, Franklin Lakes, NJ, USA) into a 50 mL conical tube. After suspending in cell medium and counting, the cells were plated onto poly-D-lysine-coated flasks (at a density of 3 × 10^4^ cells/25 cm^2^ flask). The mixture was then cultured in Dulbecco’s modified Eagle’s/F12 medium with GlutaMAX (DMEM/F12), supplemented with 10% FBS, 10,000 units/mL penicillin–streptomycin mix (ThermoFisher™, Waltham, MA, USA). The cell culture was incubated at 37 °C with 5% CO_2_. The medium was replaced after one day and supplemented with 5 ng/mL murine recombinant granulocyte and macrophage colony-stimulating factor (GM-CSF) (Sigma-Aldrich, St. Louis, MO, USA). Next, the cells were utilised for experimentation after two weeks of culture, consisting of 40% CD11b+ cells and 60% GFAP+ cells [54].

The growth of the cell cultures was monitored using a CKX53 inverted microscope (Olympus™, Warsaw, Poland). To determine the phenotype of the cells, the cells were stained during their culture (days 7 and 14 of incubation) using specific markers appropriately selected for each population analysed. The following antibodies were used: mouse anti-CD11b APC-conjugated (for microglia; ThermoFisher™), mouse anti-GFAP, Alexa Fluor 488-conjugated (for astrocytes; ThermoFisher™, Waltham, MA, USA), mouse anti-CNP (for oligodendrocytes; ThermoFisher™, Waltham, MA, USA), and mouse anti-NeuN (for neurons; ThermoFisher™, Waltham, MA, USA). The samples were analysed by confocal microscopy (FluoView FV10i and FV10-ASW 3.0 Viewer software, Olympus™, Warsaw, Poland).

### 2.2. Viruses

The mouse coronavirus (MHV, Mouse Hepatitis Virus) neuropathological strain MHV-JHM [VR-76513™, ATCC^®^, Manassas, VA, USA] was propagated in a mouse hepatocyte cell line [NCTC, CCL-9.1™, ATCC^®^, Manassas, VA, USA]. The median tissue culture infectious dose (TCID_50_) was calculated using the Spearman–Kärber method [55]. Aliquots were stored at −80 °C. All studies were conducted with the virus stock at the second passage level and a titre of 10^7.8^ TCID_50_/mL.

Sialodacryoadenitis Virus strain 682 (SDAV, courtesy of Prof. Susan Compton, Yale University, New Haven, CT, USA) was propagated in rat lung epithelium [L-2, CCL-149™, ATCC^®^, Manassas, VA, USA]. The plaque-forming units (PFU) were established via plaque assay [56]. All studies were conducted with the virus stock at the third passage level and a titre of 10^6.8^ PFU/mL.

MHV-JHM or SDAV stock was added to the cell culture and incubated for 1 h (37 °C with 5% CO_2_). The virus suspension was aspirated, and a fresh growth medium was added. Infected cultures were incubated for 224, 48, 72, 96, 168, and 672 h at 37 °C with 5% CO_2_ according to the experimental needs. In the case of 672 h of infection (1 month), cell media were added if needed to maintain the culture (about 0.5 mL for a 6-well plate and 50 µL for a 96-well plate to keep the medium level at 2 mL and 200 µL appropriately).

### 2.3. Immune Response Profiling

Conditioned cell media from mixed glial cell cultures (collected at 2, 24, 48, 72, 96, 168, and 672 (1 month) hours post infection (h p.i.)) infected with MHV and SDAV were analysed for their production of 48 cytokines, chemokines, growth factors/regulators, and soluble receptors (presented in Table 1) simultaneously for efficient immune response profiling using Luminex xMAP technology (ProcartaPlex™ Mouse Immune Monitoring Panel, 48plex, ThermoFisher™, Waltham, MA, USA). All samples were incubated in a 96-well Solid Polystyrene Microplate (Corning^®^, New York, NY, USA), and during the washing process, a magnetic 96-well separator (Ambion™ 96-well Magnetic-Ring Stand, ThermoFisher™, Waltham, MA, USA) was used following the manufacturer’s instructions.

This system contains bead set markers with varying ratios of two different fluorophores coupled to monoclonal antibodies directed against various cytokines, chemokines, growth/regulatory factors, and soluble receptors. The protein of interest was bound to a monoclonal antibody on a bead (after 24 h incubation), and a secondary detection antibody specific to the molecule of interest was added (2 h incubation). The colour-coded beads were read on a MAGPIX^®^ system (Luminex Corporation, New York, NY, USA) using two lasers. One laser is used to identify the bead and thus the protein of interest, while the other is used to detect the amount of detection agent on the bead and thus the amount of soluble protein of interest. In addition, a standard curve in duplicate has been provided by the manufacturer for each experiment. A five-parameter logistic curve was then generated, and the standard recovery was calculated using the following equation: (observed concentration/expected concentration) × 100. A desired recovery range was between 70% and 130%. Any sample outside this range was considered impure. A sample was considered positive if it exceeded the limits of detection as defined by the manufacturer’s specifications. Data acquisition was performed using the MAGPIX^®^ instrument and xMAP^®^ component software v. 4.2. A data analysis was performed using xPONENT^®^ software ver. 4.3 for Luminex instruments.

### 2.4. ELISA

An immunoenzymatic ELISA (Mouse DuoSet^®^, R&D Systems, Minneapolis, MN, USA) was used to analyse the cytokines expressed in the primary mixed glia culture infected with SDAV and MHV-JHM for 2, 24, 48, 72, 96, and 168 h. The expression levels of IL-4, IL-5, IL-6, IL-10, IL-17, and TNF alpha were analysed according to the procedure outlined by the manufacturer. Briefly, monoclonal antibodies for the tested cytokines were diluted to yield concentrations of 4 μg/mL for IL-4 and IL-10, 2 μg/mL for IL-6 and IL-17, 1 μg/mL for IL-5, and 800 ng/mL for TNF alpha. To prepare the standards, seven 2-fold serial dilutions were performed at concentrations of 1000–15.6 pg/mL for IL-4, IL-6, and IL-17 and at concentrations of 2000–31.2 pg/mL for TNF alpha, IL-5, and IL-10. Previously prepared test samples—mixed glia cells cultured in 6-well plates (uninfected cells—control and infected cells)—were transferred into 1.5 mL tubes and centrifuged for 5 min at 1200 rpm. Then, 50 μL of the supernatant (test samples) and standards was applied to 96-well plates previously coated with antibodies. The plates were incubated overnight at 4 °C. After incubation, the plates were washed with 0.05% Tween/PBS (3×). Secondary antibodies were prepared and diluted in 1% BSA/PBS to give concentrations of 250 ng/mL for IL-4 and IL-10, 75 ng/mL for IL-6, 50 ng/mL for IL-5 and IL-17, and 37.5 ng/mL for TNF alpha. Then, 50 μL of II-antibodies was applied per well and incubated for 2 h at room temperature. After this time, they were washed in 0.05% Tween/PBS (3×). In the next step, 50 μL of the streptavidin-HRP conjugate (diluted 1:40 in 1% BSA/PBS) was applied and incubated in the dark for 20 min at room temperature. After incubation with the conjugate, the plates were washed again with 0.05% Tween/PBS (3×). Then, 50 μL of a 1:1 H_2_O_2_/TMB solution was applied and incubated for 20 min at room temperature. After incubation, 50 μL of 2 M H_2_SO_4_ was applied to stop the reaction. Optical density readings at 450 nm and 570 nm were measured using a Synergy H1 plate reader (BioTek Instruments™, Winooski, VT, USA).

### 2.5. Confocal Microscopy Analysis of SDAV and MHV-JHM Infection

Control and SDAV- or MHV-JHM-infected cells (2, 24. 48, 72, 96, 168, and 672 (1 month) h p.i.) were washed with PBS and fixed with 3.7% PFA for 15 min at room temperature. After washing with PBS, the cells were incubated at room temperature with 0.1% Tween/PBS for 15 min. Next, the cells were washed twice with PBS and then incubated with BSA/PBS for 30 min at RT. For phenotyping, the following antibodies were used: mouse anti-GFAP, Alexa Fluor 488-conjugated (astrocytes, diluted 1:100 in BSA/PBS); mouse anti-CD11b APC-conjugated (microglia; diluted 1:100 in BSA/PBS); mouse anti-CNP mAb (oligodendrocytes; diluted 1:100 in BSA/PBS); and mouse anti-NeuN mAb (neurons; diluted 1:100 in BSA/PBS). Following 1 h of incubation, secondary antibody Alexa Fluor 647 (goat anti-mouse; diluted 1:250 in BSA/PBS) was added. The viral antigen was visualised by mouse anti-SARS/SARS-CoV-2 mAb specific for N protein (diluted 1:200 in BSA/PBS) and incubated for 1 h at 37 °C. After this time, the cells were washed twice in PBS, and secondary antibody AlexaFluor™ 488 (goat anti-mouse; diluted 1:500) or TexasRed (goat anti-mouse; diluted 1:1000) was added (1 h, in the dark, RT). Next, the cells were washed twice with PBS, followed by staining with phalloidin/TRITC solution (500 ng/mL) for 1h in the dark at RT to visualise the actin filaments. After incubation, the cultures were washed twice with PBS. An amount of 1 μg/mL of Bisbenzimidine/Hoechst 33258 dye was used to visualise the cell nuclei (2–3 min, RT). All the antibodies and fluorochromes were purchased from ThermoFisher™ (Waltham, MA, USA). Then, the slides were rinsed twice with PBS and once with deionised water. The slides were mounted using Prolong Gold Antifade Reagent (ThermoFisher™, Waltham, MA, USA). Image acquisition was performed with a FluoView FV10i confocal microscope (Olympus™, Warsaw, Poland), equipped with a 60× water-immersion objective. The images were processed using the FV10-ASW 3.0 Viewer software (Olympus™, Warsaw, Poland) and then converted to 24-bit TIFF files.

### 2.6. ROS Detection

The ROS levels in the mixed glial cell culture were assessed from 2, 24, 48, 72, 96, and 168 h to 1 month post-infection with SDAV and MHV-JHM. The negative control was uninfected cells, while the positive control was 1 mM H_2_O_2_-treated cells (1 min, RT). The cells were incubated in culture fluid supplemented with fluorogenic probe CellROX^®^ Green Reagent (5 µM/mL, 30 min, 37 °C; ThermoFisher™, Waltham, MA, USA), measuring oxidative stress in live cells [57]. Then, the cells were washed with PBS, stained with Hoechst 33542, and fixed with 3.7% PFA/PBS for 15 min in RT. After fixation, the cells were washed twice with PBS and mounted with Prolong Gold Antifade Reagent. The slides were analysed using a FluoView FV10i confocal microscope (Olympus™, Warsaw, Poland) equipped with a 60× water-immersion objective. The images were processed using the FV10-ASW 3.0 Viewer software (Olympus™, Poland) and then converted to 24-bit TIFF files.

The Image J/Fiji (ver. 2.14.0/1.54p, NIH Image, Bethesda, MD, USA) tool was used to quantify ROS expression. TIFF files were converted to 16-bit greyscale images. Then, the region of interest (ROI) was selected using the polygon tool for each cell, and a scale from pixels to µm was set up. This was followed by measuring the ROI of each cell to obtain specific parameters, such as the area, mean, integrated density, and raw integrated intensity. Additionally, independent blinded counts were performed around each cell ROI for manual quantitation. The obtained parameters were used to calculate Corrected Total Cell Fluorescence (CTCF) = Integrated Density − (Area of selected cell × Mean fluorescence of background readings).

### 2.7. Flow Cytometry

Flow cytometry was used to measure apoptosis/necrosis and the ROS rate in mixed glial cells after infection with MHV-JHM and SDAV (2, 24, 48, 72, 96, 168 h, and 1 month p.i.) using Dead Cell Apoptosis Kits with Annexin V for Flow Cytometry (ThermoFisher™, Waltham, MA, USA) and the CellROX™ Green Flow Cytometry Assay Kit (ThermoFisher™, Waltham, MA, USA). Infected cells (10^4^ cells/mL) and proper controls were stained according to the manufacturer’s protocols. Samples were analysed by Cytoflex LX cytometer (BD Biosciences, Franklin Lakes, NJ, USA) to specify the proportion of necrotic cells (Annexin V-FITC−/PI+), early apoptotic cells (Annexin FITC+/PI−), late apoptotic cells (Annexin V-FITC+/PI+), and non-apoptotic cells (Annexin V-FITC−/PI−), and the presence of ROS+ cells. Calculations were performed with CellQuest Pro™ analysis software ver. 5.2.1 (BD Biosciences, Franklin Lakes, NJ, USA). The experiments were performed in triplicate.

### 2.8. Statistical Analysis

The results were statistically evaluated by two-way or one-way analysis of variation (ANOVA) using the Dunnet or Tukey multiple comparison test. The non-parametric variables were assessed using the Kruskal–Wallis test with the post hoc Dunn test. All experiments were performed at least in triplicate. These analyses were performed using GraphPad Prism^TM^ version 9.4.0 (453) for macOS software (GraphPad Software Inc., San Diego, CA, USA). Statistical differences were interpreted as significant at *p* ≤ 0.05 (*), very significant at *p* ≤ 0.01 (**), highly significant at *p* ≤ 0.001 (***), and not significant at *p* > 0.05.

## 3. Results

### 3.1. SDAV Infection of Primary Mixed Microglia and Astrocyte Cells

**Figure 1:**
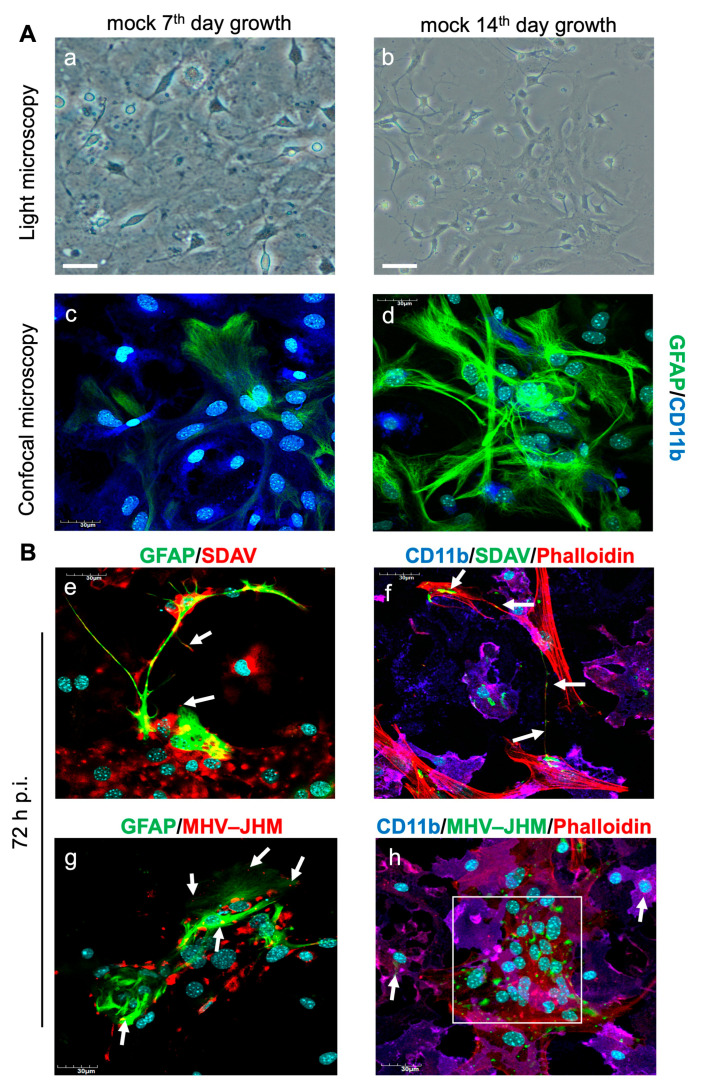
Representative images of primary astrocytes and microglia cells, uninfected (**A**) and infected with SDAV and MHV-JHM for 72 h (**B**). Cells were cultured for 14 days before each experiment. Microglia were more abundant than astrocytes during the first 7 days (**a**,**c**). After stimulation with GM-CSF, the ratio of microglia to astrocytes on the 14th day of growth was 40% to 60%, respectively (**b**,**d**). These changes were captured by a light microscope (**a**,**b**) and confocal microscope (**c**,**d**; green—GFAP (astrocytes), dark blue—CD11b (microglia), light blue—cell nuclei). After 14 days of growth, the primary mixed glial culture was infected with SDAV and MHV-JHM for 72 h and labelled using immunofluorescence (**B**). A signal corresponding to SDAV and MHV-JHM was found both in the astrocytes (**e**,**g**; green—GFAP (astrocytes), red—SDAV, MHV-JHM, light blue—cell nuclei) and microglia (**f**,**h**; dark blue—CD11b (microglia), green—SDAV, MHV-JHM, red—F-actin, light blue—cell nuclei). The white arrows show the placement of SDAV (**e**,**f**) or MHV-JHM (**g**,**h**) in the cells. The white rectangle shows syncytium formation (**h**). Microscope CKX53 (**a**,**b**) and FluoView FV10i (**c**,**d**,**B**) (Olympus™, Poland). Scale bar: 30μm.

To carry out the planned analyses, we first optimised the primary culture of astrocytes and microglia so that, after 14 days of growth, the cell ratio reached 40% microglia to 60% astrocytes (Figure 1A). The cells were infected with SDAV to check the possibility of infection of astrocytes and microglia. Observation of the 72 h infection using confocal microscopy showed that the viral antigen (red fluorescence) was initially present in astrocyte cells labelled with GFAP antibody (green fluorescence) (Figure 1(Be), white arrows). The viral antigen (green fluorescence) was detected travelling along the filopodia to reach nearby microglial cells labelled with CD11b (dark blue fluorescence) (Figure 1(Bf), white arrows). The viral antigen was present in large numbers and with high fluorescence in both cell types. We also infected mixed microglia and astrocytes with MHV-JHM as a better-known model to compare the effect of SDAV infection. After 72 h post-infection (h p.i.) the MHV-JHM antigen was visible at the cell membrane side of GFAP-labelled astrocytes (Figure 1(Bg); white arrows). In the CD11b-labelled microglia cells, the MHV antigen was located in the perinuclear region (Figure 1(Bh); white arrows). A cytopathic effect in the form of syncytium was visible (Figure 1(Bh), white rectangle).

### 3.2. FACS Analysis of Cell Death

**Figure 2:**
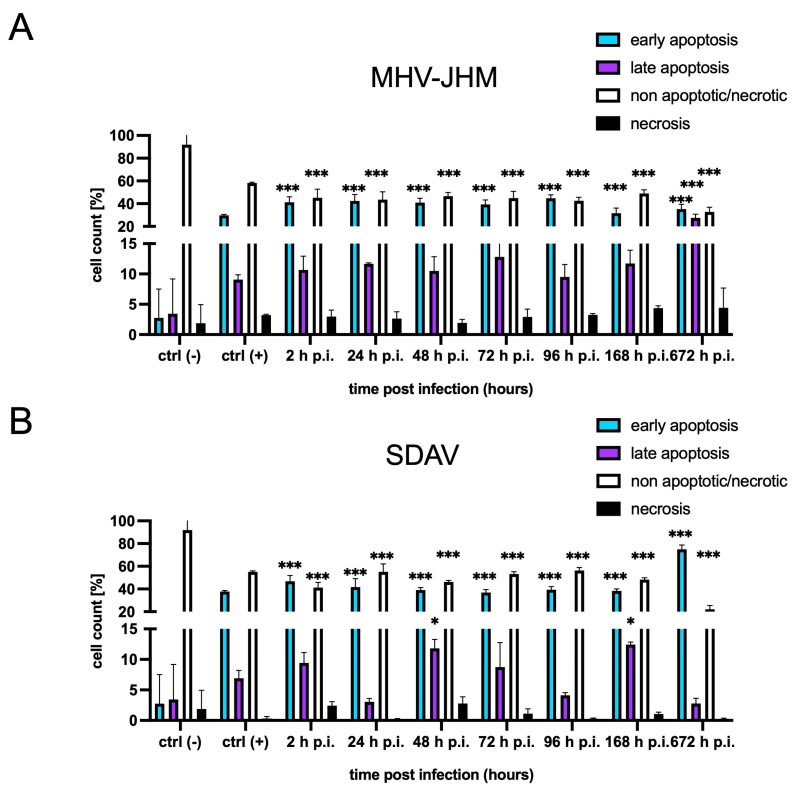
The percentage (mean  ±  SD) of early apoptotic, late apoptotic, necrotic, and non-apoptotic cells in a mixed primary microglia and astrocyte culture from 2 to 672 h p.i. with MHV-JHM (**A**) and SDAV (**B**) from three independent experiments (ANOVA, * *p*  ≤  0.05, *** *p*  ≤  0.001).

Annexin V-FITC and PI FACS analyses were performed for the differentiation and quantitative determination of the percentage of necrotic, viable, and apoptotic cells (early and late types) after infection with MHV-JHM and SDAV (Figure 2). MHV-JHM induced apoptosis in the mixed microglia and astrocytes culture within the first 2 h p.i. and persisted until 672 h p.i. (1 month). The percentage of early apoptotic cells increased significantly highly (*** *p*  ≤  0.001) from 2.75% in the uninfected control to 41.20% at 2 h p.i. and remained at a similar level until one month post infection. The late apoptotic cells increased non significantly from 3.43% in the control to 10.7% 2 h p.i. and stayed at a similar level until 168 h p.i. At 672 h p.i., late apoptosis reached 27.6% (*** *p*  ≤  0.001). Signs of cell necrosis appear the highest after 1 month of infection with MHV-JHM, reaching 4.43%, compared to the uninfected control, with 1.88% of necrotic cells (Figure 2A). However, this increase was not statistically significant. After infection with the SDAV strain, we observed a similar trend of inducing cell apoptosis. The level of early apoptotic cells increased highly significantly (*** *p*  ≤  0.001) from 2.75% in the uninfected control cells to 37.8% after 2 h p.i. and remained high until 1 month post-infection, reaching 74.9% (*** *p*  ≤  0.001) (Figure 2B). The percentage of late apoptotic cells increased statistically significantly only after 48 and 168 h p.i. (11.8% and 12.4%, respectively) compared to that in the uninfected control (3.43%). Necrosis did not occur in the case of SDAV infection in the primary mixed microglia and astrocyte cultures. A high % of viable cells remained unaffected from 2 to 168 h p.i. (55.1–48.2%); however, a high statistical decrease (*** *p*  ≤  0.001) was observed after 1 month post-infection (22.1%) compared to that in the uninfected control (91.95%) (Figure 2B).

### 3.3. ROS Expression

**Figure 3:**
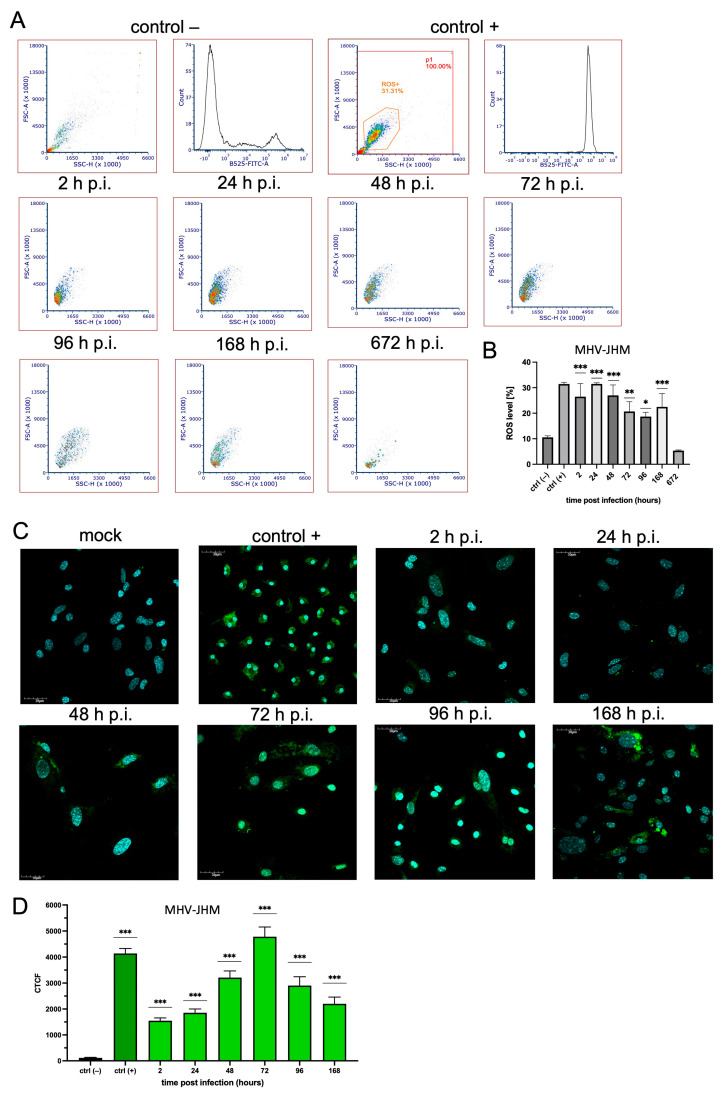
ROS production in mixed primary culture of microglia and astrocyte cells in mock and MHV-JHM-infected cells for 2–672 h. ROS levels were obtained from flow cytometry analysis using CellROX Green (ThermoFisher™) staining (**A**,**B**). Representative histograms and dot plots of ROS levels in uninfected labelled control −, uninfected labelled control + (treated with H_2_O_2_), and infected cells (**A**). The mean fluorescence intensity (MFI) of ROS-positive cells after MHV-JHM infection for 2–672 h is presented as mean ± SD (n = 10,000 cells) from three independent experiments. ANOVA; * *p* ≤ 0.05, ** *p* ≤ 0.01, *** *p* ≤ 0.001 (**B**). ROS levels were obtained from confocal microscopy analysis using CellROX Green (ThermoFisher™) staining (**C**,**D**). Representative confocal microscopy images of MHV-JHM-infected primary mixed microglia and astrocyte cells for 2–168 h. Negative control—uninfected cells; positive control—cells treated with H_2_O_2_. Cell nuclei (blue), ROS (green). Scale bar: 30 μm (**C**). Analysis of ROS fluorescence intensities performed with Fiji software (ver. 2.14.0/1.54p) described as cell total corrected fluorescence (CTCF), presented as mean ± SEM (n = 100 cells), ANOVA; *** *p* ≤ 0.001 (**D**).

**Figure 4:**
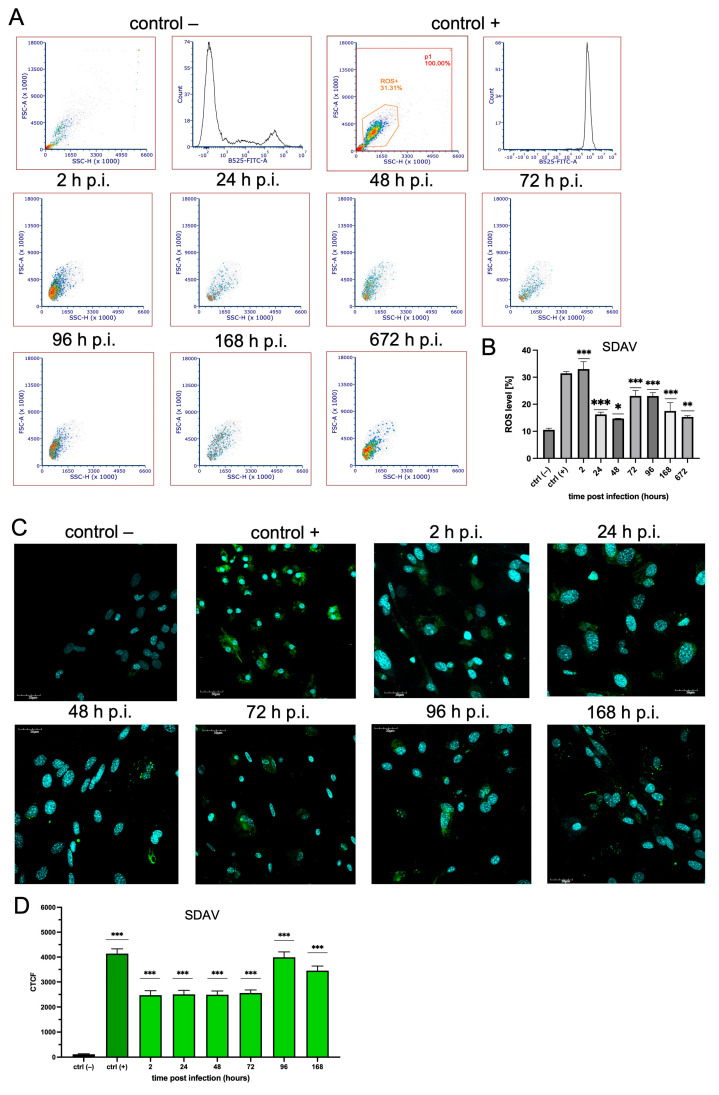
ROS production in mixed primary culture of microglia and astrocyte cells in mock and SDAV-infected cells for 2–672 h. ROS levels were obtained from flow cytometry analysis using CellROX Green (ThermoFisher™) staining (**A**,**B**). Representative histograms and dot plots of ROS levels in uninfected labelled control −, uninfected labelled control + (treated with H_2_O_2_), and infected cells (**A**). The mean fluorescence intensity (MFI) of ROS-positive cells after SDAV infection for 2–672 h is presented as mean ± SD (n = 10,000 cells) from three independent experiments. ANOVA; * *p* ≤ 0.05, ** *p* ≤ 0.01, *** *p* ≤ 0.001 (**B**). ROS levels were obtained from confocal microscopy analysis using a CellROX Green (ThermoFisher™) staining (**C**,**D**). Representative confocal microscopy images of SDAV-infected primary mixed microglia and astrocyte cells for 2–168 h. Negative control—uninfected cells; positive control—cells treated with H_2_O_2_. Cell nuclei (blue), ROS (green). Scale bar: 30 μm (**C**). Analysis of ROS fluorescence intensities performed with Fiji software (ver. 2.14.0/1.54p) described as cell total corrected fluorescence (CTCF), presented as mean ± SEM (n = 100 cells), ANOVA; *** *p* ≤ 0.001 (**D**).

We performed two experiments to investigate reactive oxygen species (ROS) release in primary mixed microglia and astrocyte cells after infection with MHV-JHM and SDAV (Figure 3A,B and Figure 4A,B). A quantitative flow cytometry analysis (% of ROS) was followed by a semi-quantitative examination of confocal microscopy imaging using corrected total cell fluorescence (CTCF). A flow cytometry analysis revealed a rapid, highly statistically significant increase in reactive oxygen species 2 h p.i. in cells infected with MHV-JHM (26.48% ± 5.12%, *** *p*  ≤  0.001) compared to the uninfected control (10.55% ± 0.61%) (Figure 3B). The highest observed ROS value was after 24 h p.i. (31.47% ± 0.46%,*** *p*  ≤  0.001) compared to that in the uninfected control (10.55% ± 0.61%) (Figure 3B). Gradually, the ROS levels decreased but were still significantly higher than the uninfected control levels, and the values were as follows: after 48 h p.i. with MHV-JHM, 26.99% ± 4.11% (*** *p*  ≤  0.001); 72 h p.i., 20.68% ± 3.84% (** *p* = 0.004); 96 h p.i., 18.68% ± 1.68% (* *p*  =  0.019); 168 h p.i., 22.48% ± 5.22% (*** *p*  ≤  0.001) compared to the uninfected control (10.55% ± 0.61%) (Figure 3B). The lowest value was noted after 672 h p.i., where the ROS level decreased insignificantly to 5.34% ± 0.22% (*p* = 0.363) compared to that for the control uninfected cells (10.55% ± 0.61%) (Figure 3B).

Cells infected with SDAV presented the highest abundance of ROS levels after 2 h p.i. The level of ROS increased highly statistically significantly (33.03% ± 2.72%, *** *p*  ≤  0.001) compared to that of the uninfected control (10.55% ± 0.61%) (Figure 4B). The values at 24 h p.i. (16.25 ± 0.85, *** *p*  ≤  0.001) and 48 h p.i. (14.71 ± 0.08, * *p*  =  0.012) slightly decreased but were still statistically significantly higher than that for the control uninfected cells (10.55% ± 0.61%) (Figure 4B). After 72 h p.i. and 96 h p.i., a highly statistically significant increase occurred at a constant level of 23.08% ± 1.96% and 23.06% ± 1.22% (*** *p*  ≤  0.001), respectively, compared to that for the uninfected control (10.55% ± 0.61%) (Figure 4B). Then, after 168 h p.i. and 672 h p.i., the ROS levels slightly decreased to 17.53 ± 3.06 (*** *p*  ≤  0.001) and 15.31 ± 0.45 (** *p*  ≤  0.005), respectively, compared to previous time points but remained statistically significantly higher than those for the uninfected control (10.55% ± 0.61%) (Figure 4B).

An analysis of the confocal images stained with a fluorogenic probe showed an evenly distributed signal of ROS in the cells after infection with both viruses (Figure 3C,D and Figure 4C,D). In the case of MHV-JHM, ROS were uniformly present throughout the cell from 2 h p.i. to 168 h p.i. (Figure 3C). After a detailed data analysis, we determined the quantitative value of the probe corresponding to the number of ROS in the form of corrected total cell fluorescence (CTCF) (Figure 3D). MHV-JHM infection triggered ROS production from 115.4 ± 216.0 in the uninfected control cells to 1547.0 ± 1091 (*** *p*  ≤  0.001), after 2 h p.i. This value remained similar until 48 h p.i., where the values highly statistically significantly increased to 3212.0 ± 2574.0 (*** *p*  ≤  0.001) compared to the uninfected control (115.4 ± 216.0). The highest rate was observed after 72 h p.i. (4784.0 ± 3700.0) compared to the uninfected control (115.4 ± 216.0).

The ROS distribution after SDAV infection of mixed microglia and astrocytes was visible on confocal images during all times post-infection. The brightest fluorescence was present after 96 and 168 h p.i. (Figure 4C). A data analysis revealed that the CTCF values were nearly similar after 2, 24, 48, and 72 h p.i. (2474.0 ± 1776.0, 2505.0 ± 1604.0, 2492.0 ± 1486.0, 2560.0 ± 1216.0; *** *p*  ≤  0.001, respectively) with SDAV, with a highly statistically significant increase compared to those for the uninfected control (115.4 ± 216.0) (Figure 4D). The highest values were recorded after 96 h p.i. (3452.0 ± 216.0) and 168 h p.i. (3991.0 ± 2177.0) compared to the uninfected control cells (115.4 ± 216.0).

### 3.4. Cytokine and Chemokine Expression

**Figure 5:**
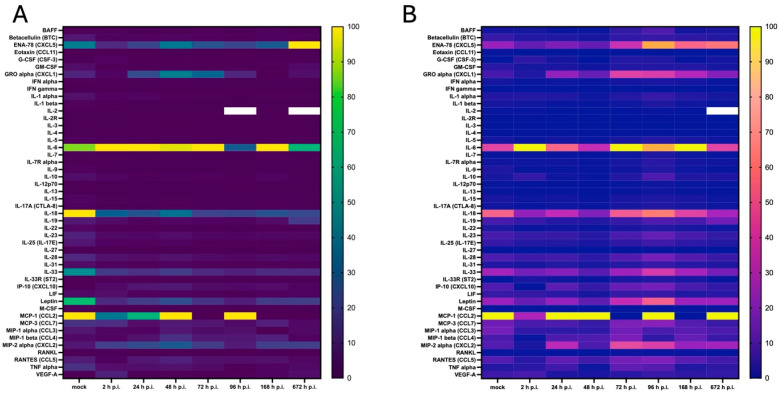
Heat map showing the cytokine concentrations secreted by primary mixed microglia and astrocyte cells after infection with MHV-JHM (**A**) and SDAV (**B**) from 2 to 672 h. Colours are assigned according to the relative, normalised scale of expression. The scale ranges from 0 to 100% (lowest to highest concentration in a row).

The levels of expressed cytokines and chemokines, as well as growth factors and soluble receptors, were measured after infection with MHV-JHM and SDAV in primary mixed microglia and astrocyte cells using multiplex technology (Luminex xMAP), analysing 48 proteins crucial for immunomodelling. The results showed changes in both viruses over the course of the one-month infection period, as visualised by a heat map. In the case of MHV-JHM infection, changes in expression (from 0% to 100%) were visible in IL-6, IL-18, IL-33, ENA-78 (CXCL5), GRO alpha (CXCL1), IP-10 (CXCL10), MCP-1 (CCL2), MIP-1 beta (CCL4), MIP-2 alpha (CXCL2), RANTES (CCL5), and TNF alpha (Figure 5A). However, SDAV infection increased the protein expression of IL-1 alpha, IL-6, IL-10, IL-18, IL-19, IL-23, IL-28, IL-33, ENA-78 (CXCL5), GRO alpha (CXCL1), IP-10 (CXCL10), MCP-1(CCL2), MCP-3(CCL3), MIP-1 alpha (CCL3), MIP-1 beta (CCL4), MIP-2 alpha (CXCL2), RANTES (CCL5), TNF alpha, and Leptin (Figure 5B).

**Figure 6:**
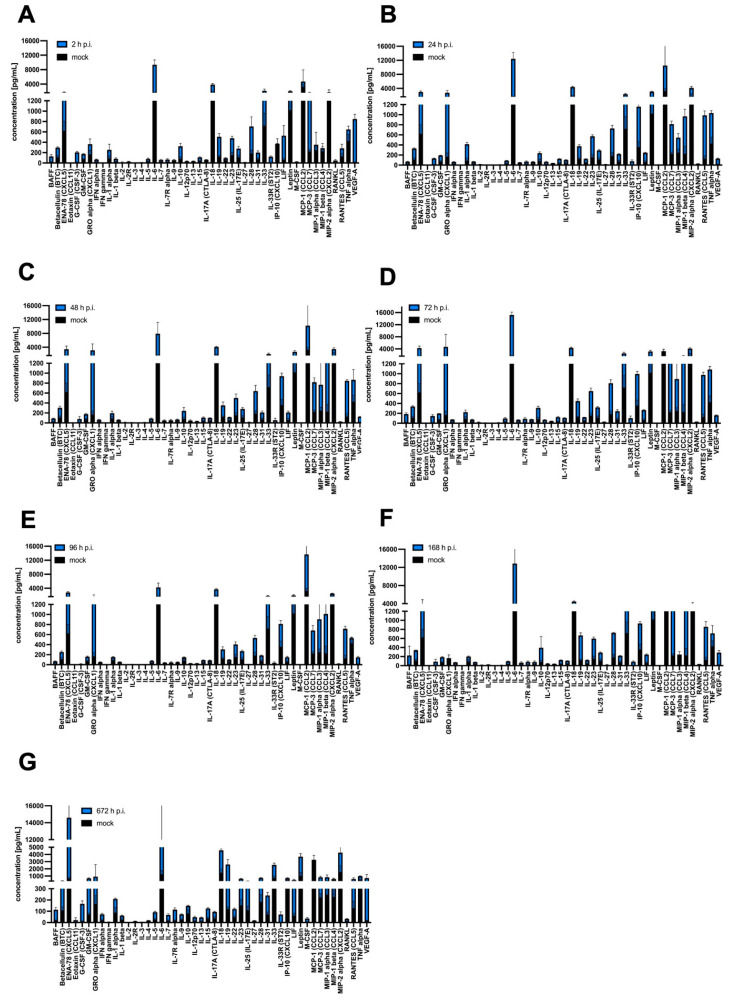
MHV-induced cytokine, chemokine, and growth factor expression [pg/mL] in infected (2–672 h) primary microglia and astrocyte cells. Bar plots indicate the concentration change of expressed 48 protein markers in a single biological replicate (n = 6 for each group), shown as mean ± SD (**A**–**G**). Statistical data are shown in Supplementary File S1.

More detailed changes in expression pattern post-infection, shown as concentration [pg/mL], were revealed by statistical analysis of the obtained protein concentration compared to the mock. After 2 h p.i. with MHV-JHM, a statistically highly significant increase compared to the mock (*** *p* ≤ 0.001) was observed for G-CSF (196.0 ± 17.73; 9.54 ± 4.65), IL-2R (23.09 ± 0.4; 3.15 ± 0.65), IL-33R (121.8 ± 14.32; 0.0), LIF (465.5 ± 194.1; 60.55 ± 21.77), and RANKL (39.88 ± 20.98; 8.35 ± 2.35). Statistically very significant (** *p* ≤ 0.01) and statistically significant (* *p* ≤ 0.05) results were obtained for BAFF (109.9 ± 32.71; 17.63 ± 4.62), IL-1 beta (57.74 ± 17.27; 23.19 ± 8.07), IL-7 (43.42 ± 9.6; 7.12 ± 3.18), IL-23 (476.3 ± 58.99; 152.2 ± 51.47), IL-28 (522.5 ± 183.3; 182 ± 63.01), IL-31 (152.4 ± 17.71; 71.12 ± 22.24), and VEGF-A (832.10 ± 93.61; 15.92 ± 2.80) (Figure 6A). At 24 h p.i., most cytokines, chemokines, and growth factors remained at the same level as at 2 h p.i., but new statistically highly significant increases were observed for IL-12 (53.41 ± 8.66; 13.77 ± 2.3), IP-10 (801.3; 80.02 ± 14.71), and RANTES (860.2; 127.0 ± 59.98). Statistically very significant (** *p* ≤ 0.01) and statistically significant (* *p* ≤ 0.05) results were obtained for GM-CSF (122.5 ± 5.96; 69.73 ± 13.45), GRO alpha (2606.0 ± 631.1; 131.6 ± 17.85), IFN gamma (4.806 ± 0.71; 1.69 ± 0.49), IL-1 alpha (327.5 ± 37.48; 87.74 ± 45.21), IL-1 beta (46.50 ± 3.75; 23.19 ± 8.07), IL-3 (3.52 ± 0.42; 1.39 ± 0.38), IL-6 (11,168.0 ± 1806.0; 912.2 ± 83.72), IL-12 (53.41 ± 8.66; 13.77 ± 2.30), Il-13 (28.25 ± 1.29; 10.29 ± 3.77), IL-15 (88.71 ± 5.97; 35.04 ± 10.80), IL-17A (65.94 ± 5.63; 27.59 ± 4.19), IL-18 (2936 ± 204.7; 1465.0 ± 456.5), IL-22 (75.52 ± 7.47; 46.10 ± 10.59), IL-27 (12.49 ± 1.36; 4.5 ± 1.11), IL-28 (544.9 ± 62.92; 182.9 ± 63.01), IL-31 (147.9 ± 12.58; 71.12 ± 22.24), IL-33 (1642.0 ± 252.6; 718.7 ± 490.9), MIP-2 alpha (2527.0 ± 416.4; 112.2 ± 48.98), and TNF alpha (608.0 ± 47.51; 221.2 ± 65.15) (Figure 6B). At 48 h p.i. MHV-JHM significantly increased the protein expression for only Eotaxin (3.4 ± 3.01; 0.0), IL-5 (61.63 ± 11.45; 25.69 ± 6.74), IL-17A (63.06 ± 6.3; 27.59 ± 35.29), IP-10 (583.3 ± 63.34; 14.71 ± 4.52), MIP-1 beta (987.3 ± 128.8; 59.89 ± 28.34), MIP-2 alpha (1892.0 ± 366.6; 112.2 ± 48.98), and RANTES (718.6 ± 23.37; 127.0 ± 59.62) (Figure 6C). However, highly significant (*** *p* ≤ 0.001) and very significant (** *p* ≤ 0.01) statistical increases were observed for almost all proteins after 72 h p.i. The highest increase in concentration was observed for ENA-78 (3661.0 ± 642.9; 621 ± 177.6), GRO-alpha (7253.0 ± 286.8; 131.6. ± 17.85), IFN-gamma (4.784 ± 0.61; 1.69 ± 0.49), IL-1 beta (48.35 ± 3.09; 23.19 ± 8.07), IL-3 (3.94 ± 0.92; 1.39 ± 0.38), IL-6 (13,941.0 ± 959.60; 912.2 ± 83.72), IL-12 (56.27 ± 7.85; 13.77 ± 2.30), IL-15 (86.99 ± 9.83; 35.04 ± 10.80), IL-18 (2866.0 ± 239.6; 1465.0 ± 456.5), IL-19 (329.0 ± 43.38; 118.7 ± 19.69), IL-23 (497.50 ± 60.68; 152.2 ± 51.47), IL-33 (1778.0 ± 405.4; 718.7 ± 490.9), LIF (205.6 ± 8.23; 60.55 ± 21.77), MCP-3 (205.6 ± 8.23; 223.5 ± 33.83), MIP-1 beta (1046.0 ± 552.80; 59.89 ± 28.34), MIP-2 alpha (2553.0 ± 366.6; 112.2 ± 48.98), and TNF alpha (559.3 ± 61.83; 221.2 ± 65.15) (Figure 6D). After 96 h p.i. with MHV-JHM, statistically very significant (** *p* ≤ 0.01) and statistically significant (* *p* ≤ 0.05) results were obtained only for MIP-1alpha (1095.0.0 ± 346.40; 246.9 ± 65.28) and MIP-1 beta (1095 ± 346.40; 59.89 ± 28.34) (Figure 6E). At 168 h p.i., protein expression was similar to that at 72 h p.i., with almost all proteins being highly significant (*** *p* ≤ 0.001), very significant (** *p* ≤ 0.01) or (* *p* ≤ 0.05) upregulated. The most significant changes in expression were observed for BAFF (109.9 ± 32.71; 17.63 ± 4.62), ENA-78 (2185.0 ± 253.7; 621 ± 177.6), IFN alpha (53.50 ± 3.38; 17.82 ± 7.19), Leptin (2135 ± 460.6; 1022 ± 1632.1), MCP-1 alpha (1095.0.0 ± 346.40; 246.9 ± 65.28), MCP-3 (1164.0 ± 164.90; 223.5 ± 33.83), MIP-1 beta (1532 ± 967.40; 59.89 ± 28.34), MIP-2 alpha (2163 ± 366.6; 112.2 ± 48.98), RANKL (205.4 ± 207.10; 8.35 ± 2.35), and VEGF-A (273.20 ± 45.16; 15.92 ± 2.80) (Figure 6F). Finally, after one month p.i. with MHV-JHM, all of the cytokines, chemokines, and growth factors were still significantly increased compared to the mock. The greatest changes were observed for ENA-78 (3237.0 ± 1024.0; 621 ± 177.6), G-CSF (156.20 ± 26.38; 9.54 ± 4.65), GM-CSF (616.50 ± 66.73; 69.73 ± 13.55), IL-6 (13,941.0 ± 83.72; 15,590 ± 4491), IL-33 (1827.0 ± 262.5; 718.7 ± 490.9), MIP-2 alpha (3361 ± 378.81; 112.20 ± 48.98), and VEGF-A (695.00 ± 516.20; 15.92 ± 2.80) (Figure 6G).

**Figure 7:**
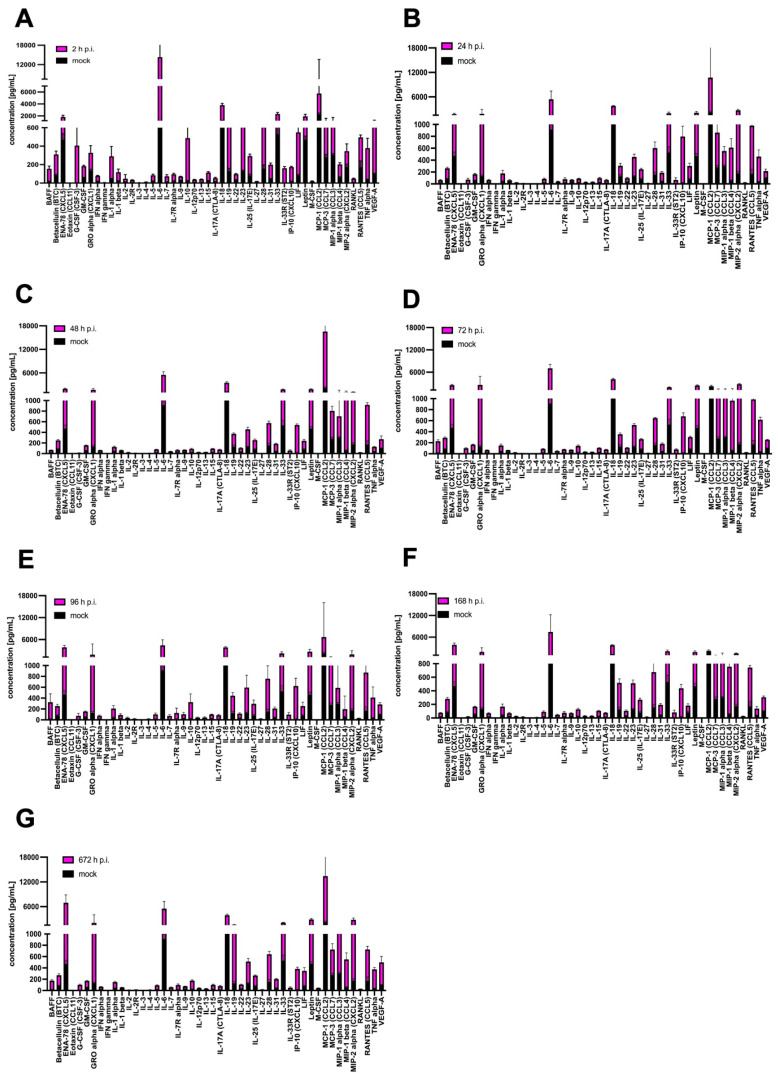
SDAV-induced cytokine, chemokine, and growth factor expression [pg/mL] in infected (2–672 h) primary microglia and astrocyte cells. Bar plots indicate a concentration change in expressed 48 protein markers in a single biological replicate (n = 6 for each group), shown as mean ± SD (**A**–**G**). Statistical data are shown in Supplementary File S1.

SDAV infection of primary microglia and astrocytes revealed slightly different protein expression. After 2 h p.i., a statistically highly significant increase compared to mock (*** *p* ≤ 0.001) was observed for betacellulin (224.90 ± 35.99; 85.02 ± 7.39), G-CSF (402.8 ± 275.90; 2.81 ± 1.39), IFN alpha (59.54 ± 6.21; 18.33 ± 3.44), IFN gamma (4.58 ± 0.58; 1.30 ± 0.08), IL-1 alpha (245.5 ± 6.81; 44.02 ± 6.81), IL-1 beta (93.39 ± 34.13; 23.92 ± 3.87), IL-3 (3.78 ± 0.49; 0.99 ± 0.14), IL-6 (13,400.00 ± 3749; 912.20 ± 83.72), IL-7 (62.24 ± 17.92; 10.55 ± 2.37), IL-10 (460.5 ± 183.40; 26.15 ± 1.92), IL-13 (33.79 ± 3.08; 7.41 ± 0.72), IL-15 (86.93 ± 9.84; 26.82 ± 0.87), IL-23 (476.30 ± 58.99; 129.90 ± 9.56), IL-28 (604.80 ± 48.53; 155.40 ± 41.10), IL-33 (1794.00 ± 256.00; 529.40 ± 97.05), Leptin (1453.00 ± 348.10; 468.40 ± 41.09), MCP-3, (1030 ± 254.10; 277.80 ± 32.41), RANKL (41.35 ± 5.99; 6.68 ± 0.68), and VEGF-A (817.60 ± 366.0; 101.00 ± 5.11). Statistically very significant (** *p* ≤ 0.01) and statistically significant (* *p* ≤ 0.05) results were obtained for BAFF (136.60 ± 29.64; 17.80 ± 2.72), IL-4 (8.51 ± 0.75; 3.48 ± 0.22), IL-12 (28.56 ± 3.76; 7.54 ± 1.14), IL-18 (2659 ± 285.70; 1148 ± 111.7), IL-19 (477.6 ± 66.11; 126.4 ± 32.87), IL-27 (13.26 ± 1.55; 4.43 ± 0.66), IL-31 (152.40 ± 17.71; 46.57 ± 1.76), M-CSF (15.99 ± 4.04; 3.40 ± 0.46), and TNF alpha (340.30 ± 106.70; 38.23 ± 4.62) (Figure 7A). At 24 h p.i. with SDAV, the concentration of most cytokines, chemokines, and growth factors decreased, but only a few remained significantly elevated: Eotaxin (1.39 ± 0.70; 0.00 ± 0.00, (* *p* ≤ 0.05)), IL-6 (4509.00 ± 2010.00; 912.20 ± 83.72, (*** *p* ≤ 0.001)), IP-10 (780.5 ± 173.78; 16.34 ± 3.96, (** *p* ≤ 0.01)), RANTES (810.10 ± 131.30; 170.10 ± 70.05, (** *p* ≤ 0.01)), and TNF alpha (423.10 ± 110.20; 38.23 ± 4.62, (** *p* ≤ 0.01)) (Figure 7B).After 48 h p.i. with SDAV, only IL-17A (59.87 ± 6.83; 15.20 ± 1.61), MIP-1 beta (982.2 ± 310.20; 58.72 ± 9.73), and RANTES (748.9 ± 39.83; 170.10 ± 70.05, (** *p* ≤ 0.01) showed significant increases in protein expression (Figure 7C). Interestingly, highly significant (*** *p* ≤ 0.001) and very significant (** *p* ≤ 0.01) statistical increases were observed for almost all proteins after 72 h p.i. The greatest change in concentration was observed for BAFF (208.80 ± 28.49; 17.80 ± 2.72), Eotaxin (5.69 ± 1.05; 0.00 ± 0.00), G-CSF (97.67 ± 8.80; 2.81 ± 1.39), GRO alpha (4060 ± 955.50; 131.60 ± 17.85), IFN gamma (4.03 ± 0.34; 1.30 ± 0.08), IL-6 (6126.00 ± 1062.00; 912.2 ± 83.72), IL-12 (28.43 ± 2.71; 7.54 ± 1.14), IL-15 (79.38 ± 5.77; 26.82 ± 1.95), IL-17A (61.66 ± 3.50; 15.20 ± 1.61), IL-18 (2966.00 ± 221.00; 1148 ± 111.70), IL-22 (75.92 ± 8.65; 33.11 ± 4.46), IP-10 (661.90 ± 64.25; 16.34 ± 3.96), Leptin (1940.00 ± 169.20; 468.40 ± 41.09), MCP-3 (922.5.50 ± 303.30; 277.80 ± 32.41), MIP-1 beta (1131.0 ± 386.40; 58.72 ± 9.73), MIP-2 alpha (2627.00 ± 164.70; 172.2 ± 79.46), RANTES (815.20 ± 105.80; 170.10 ± 70.05), and TNF alpha (577.80 ± 45.75; 38.23 ± 4.62). Statistically significant changes in expression were also observed for IL-3 (3.26 ± 0.55; 0.99 ± 0.14), IL-9 (43.28 ± 2.39; 29.70 ± 2.48), IL-10 (117.3 ± 16.85; 26.15 ± 1.92), IL-23 (391.70 ± 26.09; 129.9 ± 9.56), IL-28 (491.80 ± 12.62; 155.40 ± 41.10), and LIF (220.30 ± 17.15; 81.76 ± 10.92) (Figure 7D). After 96 h p.i. with SDAV, the protein expression pattern remained similar to that at 72 h p.i., but only a few proteins changed. Statistically highly significant (*** *p* ≤ 0.001) increases were observed only for BAFF (305.20 ± 153.50; 17.80 ± 2.72), IL-7 (60.98 ± 23.70; 10.55 ± 2.37), IL-9 (72.36 ± 34.69; 29.70 ± 2.48), IL-10 (298.70 ± 151.00; 26.15 ± 1.92), IL-13 (36.03 ± 12.18; 7.41 ± 0.72), and Il-17A (70.36 ± 11.91; 15.20 ± 1.61). Other cytokines and chemokines with very significant increases (** *p* ≤ 0.01) were ENA-78 (3385.00 ± 562.70; 469.00 ± 67.43), Eotaxin (2.52 ± 1.63; 0.00 ± 0.00), IFN gamma (4.03 ± 0.34; 1.30 ± 0.08), IL-1β (63.32 ± 22.31; 23.92 ± 3. 87), IL-4 (12.34 ± 7.12; 3.48 ± 0.22), IL-12 (32.58 ± 10.96; 7.54 ± 1.14), IL-28 (599.30 ± 240.50; 155.40 ± 41. 10), IL-31 (159.50 ± 27.89; 46.57 ± 1.76), leptin (2202.00 ± 641.40; 468.40 ± 41.09), and MCP-3 (770.60 ± 229.90; 277.80 ± 32.41). Finally, other proteins were elevated statistically significantly (* *p* ≤ 0.05): IFN alpha (55.02 ± 13.29; 18.33 ± 3.44), IL-5 (78.99 ± 24.36; 19.70 ± 2.57), IL-18 (2673 ± 222.80; 1148 ± 111.7), IL-19 (317.1 ± 55.83; 126.4 ± 32.87), Il-23 (463.60 ± 226.10; 129.9 ± 9.56), IL-33 (1652.00 ± 448.70; 529.40 ± 97.05), IP-10 (605.70 ± 141.80; 16.34 ± 3.96), RANKL (41.35 ± 5.99; 6.68 ± 0.68), and TNF alpha (371.10 ± 195.70; 38.23 ± 4.62) (Figure 7E). At 168 h p.i., protein expression was only very significantly increased (** *p* ≤ 0.01) or significantly increased (* *p* ≤ 0.05) for a few datasets. The most significant change in expression was found for BAFF (52.69 ± 6.21; 17.80 ± 2.72), ENA-78 (3410.00 ± 484.40; 469.00 ± 67.43), GM-CSF (109.80 ± 8.622; 6.51 ± 4.46), GRO alpha (2232 ± 751.60; 131.60 ± 17.85), IFN alpha (51.57 ± 8.68; 18.33 ± 3.44), IL-1 alpha (123.70 ± 36.74; 44.02 ± 6.81), IL-5 (70.87 ± 13.67; 19.70 ± 2.57), IL-15 (86.93 ± 9.84; 26.82 ± 1.95), IL-17A (59.93 ± 5.85; 15.20 ± 1.61), IL-19 (391.30 ± 59.78; 126.4 ± 32.87), IL-23 (381.60 ± 49.21; 129.9 ± 9.56), IL-27 (11.96 ± 3.37; 4.43 ± 0.66), IL-33 (1579.00 ± 317.60; 529.40 ± 97.05), MIP-1 beta (696.50 ± 142.60; 58.72 ± 9.73), and RANTES (572.30 ± 31.60; 670.6 ± 70.05) (Figure 7F). Finally, after one month p.i. with SDAV, most of the cytokines, chemokines, and growth factors were significantly increased compared to the mock. The greatest highly significant (*** *p* ≤ 0.001) changes were observed for ENA-78 (6467.00 ± 1908.00; 469.00 ± 67.43), IL-4 (3.48 ± 0.22; 10.41 ± 1.88), IL-12 (35.38 ± 6.38, 7.54 ± 1.14), IL-19 (1456.00 ± 69.00; 126.40 ± 32.87), IL-27 (14.77 ± 1.49; 4.43 ± 0.66), Leptin (2426 ± 216.1; 468.4 ± 41.09), MIP-2 alpha (2645.00 ± 404.4; 172.2 ± 79.46), RANKL (21.84 ± 1.39; 6.68 ± 0.68), and VEGF (396.6 ± 106.40; 101.00 ± 5.11) (Figure 7G).

**Figure 8:**
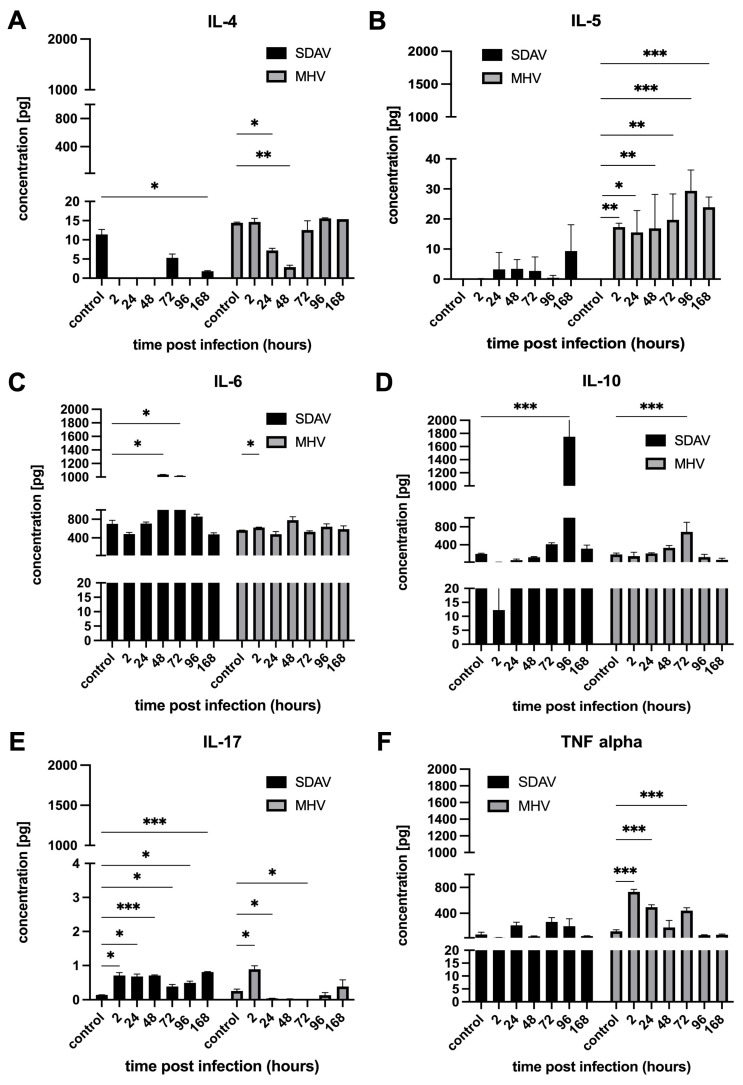
Concentration of selected cytokines produced by primary mixed microglia and astrocytes during SDAV and MHV-JHM (2–168 h) infection, analysed by ELISA. Control cells were not infected. Results are presented as the mean of calculated concentration [pg/mL] ± SD. Statistics were determined using ANOVA, * *p* ≤ 0.05, ** *p* ≤ 0.01, *** *p* ≤ 0.001, (**A**–**F**).

The next step was performing a classical ELISA for representative cytokines important for viral infection—IL-4, IL-5, IL-6, IL-10, IL-17, and TNF alpha. The anti-inflammatory cytokine IL-4 levels were significantly decreased in SDAV during all time points compared to the control (11.42 ± 1.28, * *p* ≤ 0.05). For MHV-JHM, a significant decrease was observed for 24 h p.i. (7.23 ± 0.55, * *p* ≤ 0.05) and 48 h p.i.; (2.89 ± 0.51, ** *p* ≤ 0.01) compared to the control (14.43 ± 0.17). A nonsignificant increase was only noted at 168 h p.i. with MHV-JHM (15.39 ± 0.55) compared to the control (14.43 ± 0.17). In the case of the pro-inflammatory cytokine IL-5, MHV-JHM induced a statistically significant increase compared to the uninfected cells at all time points. The highest value was observed at 96 h p.i. (29.36 ± 6.91, *** *p* ≤ 0.001) and 168 h p.i. (23.93 ± 3.39, *** *p* ≤ 0.001) compared to the control (0.00 ± 0.00). After infection with SDAV, the increase in cytokine concentrations was visible after 24, 48, 72, and 168 h, but the results were not statistically significant compared to those of the control cells. (Figure 8B). The level of pro-inflammatory cytokine IL-6 changed after infection with both viruses compared to the mock. A statistically significant increase was observed after 48 h p.i. (1035.92 ± 3.64, * *p* ≤ 0.05) and 72 h p.i. (1017.92 ± 1.80, * *p* ≤ 0.05) after infection with SDAV compared to the control (701.62 ± 77.37). After infection with MHV-JHM, a statistically significant increase was observed after 2 h p.i. (624.24 ± 8.18; * *p* ≤ 0.05) compared to the control (564.98 ± 1.54) (Figure 8C). The anti-inflammatory cytokine IL-10 was statistically highly significant after 96 h p.i. (1748.76 ± 398.05, *** *p* ≤ 0.001) with SDAV and after 72 h p.i. (687.33 ± 214.67, *** *p* ≤ 0.001) with MHV-JHM (Figure 8D) in comparison to both controls (199.97 ± 14.77, 184.91 ± 32.58, respectively). The pro-inflammatory cytokine IL-17 levels were statistically increased at all time points after SDAV infection. The highest levels were observed at 2 h p.i. (0.71 ± 0.09, * *p* ≤ 0.05), 48 h p.i. (0.71 ± 0.02, *** *p* ≤ 0.001), and 168 h p.i. (0.81 ± 0.012, *** *p* ≤ 0.001) compared to the control (0.14 ± 0.003). MHV-JHM infection induced a significant change in expression after 2 h p.i. (0.89 ± 0.11, * *p* ≤ 0.05), 24 h p.i. (0.04 ± 0.01, * *p* ≤ 0.05), and 72 h p.i. (0.13 ± 0.01, * *p* ≤ 0.05) compared to the control (0.26 ± 0.05) (Figure 8E). During SDAV infection, an increase in TNF alpha expression was observed, but it was not statistically significant compared to the control (71.42 ± 36.38). A statistically highly significant increase was observed after 2 h p.i. (731.33 ± 40.74, *** *p* ≤ 0.001), 24 h p.i. (495.08 ± 36.58, *** *p* ≤ 0.001), and 72 h p.i. (440.51 ± 44.23, *** *p* ≤ 0.001) with MHV-JHM compared to the control (119.62 ± 26.20) (Figure 8F).

## 4. Discussion

There are many studies concerning coronaviral infection outcomes in the brain conducted on patients suffering from long-COVID syndrome. However, we still seek answers to the underlying molecular reasoning behind serious health complications [58,59,60,61]. More and more research is focused on analysing the immune reaction, seeing abnormalities in cytokine/chemokine release as a main cause [62]. It is established that several viruses can induce cytokine storm syndrome/cytokine release syndrome, including, but not limited to, SARS-CoV-2, Human Immunodeficiency Virus (HIV) (including both AIDS infection and secondary infection-induced infections/malignancies), Epstein–Barr Virus (EBV), Human Herpesvirus 6 (HHV-6), Cytomegalovirus (CMV), various haemorrhagic fever viruses, and influenza viruses [63,64,65]. Studying how viruses affect glial cell function to trigger specific immune responses is important for creating targeted therapeutic strategies. Understanding the importance of further research on this topic, we have analysed two animal coronavirus models—MHV-JHM and SDAV—to answer the unknown molecular mechanism of long-COVID syndrome. Both viruses can infect rodents’ central nervous system (CNS) [50,66] and cause neurodegeneration [49,50,53,67]. However, little is known about their infection effect on microglia and astrocytes. Most research has been conducted on the MHV-A59 strain [31,68,69,70]. Some recent studies on MHV-JHM presented the roles for microglia in both demyelination and remyelination in vivo [71,72,73,74]. However, there are no data regarding SDAV. Considering this, MHV-JHM and SDAV constitute a good model that we used to investigate the effects of infection on primary mixed microglia and astrocyte cultures. We focused on characterising the type of cell death, reactive oxygen species (ROS) production, and cytokine/chemokine expression in response to infection with each virus. It is worth underlining that such research was conduct for the first time to reveal the effect of infection on glial cells and enrich the knowledge about SDAV infection in the CNS.

As mentioned, MHV-JHM was proven to infect microglia in vitro and in vivo [31,68,69,70]. However, any data considering SDAV replication in this kind of cell have not yet been described. We demonstrated that SDAV could effectively infect primary mixed microglia and astrocyte cultures. The results showed that viral antigens were present in both cell types (Figure 1(Be)). Confocal microscopy revealed that the viral antigens were initially present in the astrocytes and later detected in the microglial cells (Figure 1(Bf), white arrows).

Both MHV-JHM and SDAV induced apoptosis in the mixed glial cultures, as evidenced by Annexin V-FITC and PI FACS analyses. MHV-JHM triggered early apoptosis within 2 h p.i., sustaining increased levels throughout the one-month infection (Figure 2A). Cells infected with SDAV displayed a similar early apoptotic response, significantly increasing late apoptosis at 48 and 168 h p.i. (Figure 2B). Notably, necrosis was minimal in the SDAV-infected cells, contrasting with a slight increase observed after one month p.i. with MHV-JHM (Figure 2A,B). In comparison, previous studies confirmed that MHV-JHM induces caspase-mediated apoptosis in oligodendrocytes and the damage of myelin sheath in the CNS. This suggests one of the underlying mechanisms for the pathogenesis of MHV-induced demyelinating diseases in animals, along with the activation of the Fas signalling pathway [75,76]. On the other hand, SARS-CoV-2 infection in microglia (HMC3 cell line) induced the gene expression of antiviral immune and ER stress responses in the early phase of infection and apoptosis in the late phase of infection by intrinsic and death receptor (DR)-mediated apoptosis [77]. Data concerning SDAV apoptosis patterns have not been described so far.

A possible link to the high early apoptosis percentage after infection with MHV-JHM and SDAV can be due to ROS production by microglia and astrocytes as cell defensive mechanisms. As Villalpando-Rodriguez and Gibson (2021) suggested, the level and the type of ROS determine the ability of cells to undergo cell death [78]. Our study demonstrated a rapid and significant increase in ROS production in response to both MHV-JHM and SDAV infection, confirmed by flow cytometry and a data analysis of confocal images (Figure 3 and Figure 4). Infection with MHV-JHM induced a significant increase in ROS production levels at 2 h p.i., remaining significantly higher than uninfected control cells until one week of infection (Figure 3A,B). SDAV infection induced ROS production as early as 2 h p.i. and maintained elevated levels throughout the infection (up to 1 month) compared to the control (Figure 4A,B). The confocal microscopic analysis demonstrated the expression of ROS in the infected cells, and a data analysis revealed a highly significant increase in CTCF levels. After infection with MHV-JHM, the highest ROS fluorescence was observed at 72 h p.i., whereas for SDAV, it was observed at 96 h p.i. (Figure 3C,D and Figure 4C,D). The levels of ROS fluorescence in the cells infected with MHV-JHM at 72 h p.i. and with SDAV at 96 h p.i. and 168 h p.i. were nearly as high or higher than in uninfected positive control cells treated with H_2_O_2_ (Figure 3D and Figure 4D). The constant ROS production observed in our study suggests a prolonged state of oxidative stress in the infected mixed primary microglia and astrocyte cells, potentially exacerbating neuroinflammation and contributing to encephalitis, mimicking long-COVID effect.

A high-throughput Luminex analysis revealed crosstalk of cytokine and chemokine signalling following MHV-JHM and SDAV infection of mixed primary microglia and astrocytes (Figure 5, Figure 6, Figure 7 and Figure 8). At the early stages of infection, at 2 h p.i., SDAV infection induced a much broader and more pronounced cytokine response compared to MHV-JHM (Figure 6A and Figure 7A). We have shown that SDAV infection triggers statistically significant increases in the expression levels of betacellulin, G-CSF, IFN alpha, IFN gamma, IL-1 alpha, IL-1 beta, IL-3, IL-6, IL-7, IL-10, IL-13, IL-15, IL-23, IL-28, IL-33, Leptin, MCP-3, RANKL, and VEGF-A. At the later replication phase (from 72 h p.i.), both viruses caused a broad increase in cytokine and chemokine expression; however, SDAV generally maintained a higher overall level of cytokine expression compared to MHV-JHM (Figure 6D–G and Figure 7D–G). By one-month p.i., the most significant increases for MHV-JHM were observed for ENA-78, G-CSF, GM-CSF, IL-6, IL-33, MIP-2 alpha, and VEGF-A (Figure 6G). Notably, SDAV uniquely induced cytokines like IL-1 alpha, IL-10, IL-19, IL-23, and IL-28, which were not significantly elevated in MHV-JHM infection (Figure 7G).

Both viruses induced the expression of cytokines like IL-6, IP-10, and TNF alpha, but the temporal dynamics of their expression varied. MHV-JHM exhibited a more sustained increase in certain cytokines, such as IL-6 and IL-33, throughout the one-month period. SDAV presented a more transient response, showing a decrease in most cytokines at 24 h p.i. (Eotaxin, IL-6, IP-10, RANTES, and TNF alpha), followed by a resurgence at later time points. Recent studies on SARS-CoV-2 revealed that COVID-19-associated cytokine storms (CSs) show elevated levels of IL-1 beta, IL-6, CXCL10, TNF alpha, IFN gamma, MIP 1 alpha, and 1 beta, as well as MCP-1, GM-CSF, VEGF, and IL-10 [79,80,81]. A comparison of the present study’s findings with those of related research involving MHV-A59 reveals the upregulation of several cytokines in type I astrocytes, including IL-1 alpha, IL-1 beta, IL-2, IL-15, IL-13, IL-17, all three interferons, and TNF. The absence of induction of anti-inflammatory cytokines, such as IL-4 and IL-10, due to coronavirus infection may provide a potential explanation for the heightened inflammatory response observed during a “cytokine storm” [31]. The same pattern of increased pro-inflammatory cytokine secretion and depletion of anti-inflammatory cytokines was seen in our results, both in the case of MHV-JHM and SDAV. Consequently, there is a high probability of the occurrence of cytokine release syndrome (CRS) in mixed primary microglia and astrocyte cells. It is known that reactive astrocytes and microglia (pathogen-induced) can express and secrete crucial cytokines such as IL-1 beta, IL-6, TNF alpha, IL-18, TGF beta, and IL-10 after acute tissue injury [21,82]. Karki et al. (2021) established a new paradigm for defining the mechanism of cell death through “PANoptosis” induced by cytokine-mediated inflammation. Their research proved that increased TNF alpha and IFN gamma levels resulted in cytokine shock in mice and mirrored cytokine storm syndrome in COVID-19 patients [83]. The increased expression of these cytokines in our study can also be associated with this mechanism, but in our case, the triggering agent is a viral infection.

Both viruses induced a significant increase in key chemokines. These included IP-10, RANTES, MIP-2 alpha, ENA-78, MCP-1 alpha, and RANKL for MHV-JHM and Eotaxin, ENA-78, Gro alpha, IP-10, RANTES, RANKL, MCP-3, and MIP-1 alpha for SDAV. In comparison, the study by Miura et al. (2007) showed that alveolar type I cells infected with RCoV-P (Parkers Rat Coronavirus) or SDAV induced the expression of chemokines, CINC-2 (GRO), CINC-3 (MIP-2), LIX (ENA-78), MIP-3 alpha, and fractalkine at both 6 h and 24 h after inoculation [84]. In a separate study by Funk et al. (2009), the levels of inflammatory cytokines IL-1 alpha, IL-1 beta, TNF alpha, IFN gamma, and IL-6 in bronchoalveolar lavage fluid (BALF) did not increase significantly following SDAV infection. However, chemokines that were upregulated following SDAV infection of the airway and alveolar epithelial cells for 12 days were identified. The chemokines that were found to be significant in this study were MCP-1, LIX (ENA-78), and IP-10 [85]. Chemokines can have multiple functions in the CNS, from crucial ones like modulation of the activity of microglia and astrocytes to maintaining the health of neurons or being involved in common response mechanisms to viral infection. Elevated levels of these chemokines are often correlated with more severe disease outcomes. For instance, higher levels of IP-10 and MIP-2 alpha have been associated with increased disease severity in COVID-19 patients [86,87]. Notably, IP-10 expression can regulate the synthesis of other biomolecules. These include MCP-3, MCP-1, MIP1 alpha, RANTES, MIP-2 alpha, GRO alpha, IL-7, IL-6, and IFN gamma [88,89,90,91,92,93]. This correlation has been observed in our results for SDAV at all time points and for MHV-JHM at 24, 48, 72, and 168 h p.i. (Figure 6 and Figure 7). Similarly, persisting ENA-78 and MIP-2 alpha levels throughout the infection of both viruses may indicate amplification of innate immunity by microglia through the recruitment of neutrophils and monocytes [18,94].

The high level of IL-6 probably induced IL-10 and VEGF-A expression for possible tissue repair at the initial and late stages of infection (Figure 6, Figure 7 and Figure 8). On the other hand, we observed at 2, 96, and 672 h p.i. with SDAV an IL-4 synergy with IL-5 to possibly dampen neurotoxic Th1/Th17 responses (Figure 7) [18,21]. Significantly high levels of betacellulin and RANKL expression after one month of infection with SDAV and MHV-JHM may contribute to astrocyte-mediated repair and T-cell regulation (Figure 6 and Figure 7) [18,21]. Constantly elevated TNF-alpha, IL-1 beta, IL-1 alpha, IFN gamma, and IL-6 levels throughout infection with both viruses (Figure 6, Figure 7 and Figure 8) might be linked to sustained apoptosis (Figure 2). As described before, microglial-derived TNF alpha and IL-1 beta secretion activated astrocytes via NF-κB and MAPK pathways, inducing nitric oxide (NO) production through inducible nitric oxide synthase (iNOS), which may trigger astrocyte apoptosis [11,95,96]. Additionally, persisting IL-1 alpha levels promoted possible reactive astrogliosis via high ROS generation (Figure 3B,D and Figure 4B,D), as was also confirmed by others [11,96].

In studies conducted on COVID-19 patients, proinflammatory cytokines like IFN gamma, IL-1, 2, 6, 10, IP-10, (MCP-1), and (GM-CSF) were elevated [97]. The mechanism behind the excessive production of cytokines is that a deregulated immune system causes vascular leakage, leading to an increase in the permeability of the BBB, allowing the virus to enter the CNS, causing neurotoxicity, neuroinflammation, and neurodegeneration by apoptosis, cell lysis, or disrupting transcriptional pathways [98]. Additionally, the phenotype of glial cells can change due to viral infection into activated astrocytes and microglial cells, which can be neurotoxic (A1 phenotype and M1 phenotype of microglial cells) or neuroprotective (A2 phenotype and M2 phenotype of microglial cells) [99]. Moreover, it has been shown that the presence of reactive proinflammatory microglia secreting IL-1 alpha, IL-1 beta, IL-6, and TNF alpha or exposure to PAMP/DAMP can induce the expression of proinflammatory genes in astrocytes that trigger neuroinflammation and neurodegeneration. Moreover, A1 astrocytes secrete CCL-2, CX3CL1, CXCL10, GM-CSF, and IL-1, activating pro-inflammatory microglia [44,99,100,101,102].

## 5. Conclusions

This study demonstrated, for the first time, that SDAV can effectively infect primary murine microglia and astrocyte cells. Both viruses induced a rapid and significant increase in ROS production, with MHV-JHM causing an initial rise, followed by a gradual decrease. At the same time, SDAV maintained elevated levels throughout the infection. This prolonged oxidative stress may suggest a contribution to neuroinflammation and might mimic long-COVID effects. SDAV infection induced a broader and more pronounced early cytokine response than MHV-JHM. Both viruses caused a broad increase in cytokine and chemokine expression at later stages, with SDAV generally maintaining higher levels. Our study thus provides novel insights into the effects of MHV-JHM and SDAV infection on primary mixed microglia and astrocyte cultures, offering potential molecular explanations for cytokine release syndrome (CRS). It is also important to recognise that even mild inflammation of the cells that act as a protective coating for neurons can interfere with proper functioning of the central nervous system.

## Figures and Tables

**Table 1:** List of the soluble immune factors included in this study.

Type	Target List
Cytokines:	BAFF, G-CSF (CSF-3), GM-CSF, IFN alpha, IFN gamma, IL-1 alpha, IL-1 beta, IL-2, IL-3, IL-4, IL-5, IL-6, IL-7, IL-9, IL-10, IL-12p70, IL-13, IL-15/IL-15R, IL-17A (CTLA-8), IL-18, IL-19, IL-22, IL-23, IL-25 (IL-17E), IL-27, IL-28, IL-31, IL-33, LIF18, M-CSF, RANKL, TNF alpha
Chemokines:	ENA-78 (CXCL5), Eotaxin (CCL11), GRO alpha (CXCL1), IP-10 (CXCL10), MCP-1 (CCL2), MCP-3 (CCL7), MIP-1 alpha (CCL3), MIP-1 beta (CCL4), MIP-2, RANTES (CCL5)
Growth factors/regulators:	Betacellulin (BTC), Leptin, VEGF-A
Soluble receptors:	IL-2R, IL-7R alpha, IL-33R (ST2)

## Data Availability

All original contributions presented in this study are included in this article and its Supplementary Materials. Further inquiries can be directed to the corresponding authors.

## Supplementary Materials

The following supporting information can be downloaded at https://www.mdpi.com/article/10.3390/cells14090637/s1; Figures S1 and S2: Statistical analysis of cytokine profile for Figure 6 and Figure 7. Figure S1. MHV-induced cytokine, chemokine, receptor and growth factor expression [pg/mL] in infected (2–672 h) primary microglia and astrocyte cells. Each dot plot represents the concentration of one protein from ProcartaPlex™ Mouse Immune Monitoring Panel, 48plex. Results are shown as mean ± SD, (n = 6 for each time point). Statistics were determined using a Kruskal—Wallis Test with a Dunn Test post hoc, * *p* ≤ 0.05, ** *p* ≤ 0.01, *** *p* ≤ 0.001. Figure S2. SDAV-induced cytokine, chemokine, receptor and growth factor expression [pg/mL] in infected (2–672 h) primary microglia and astrocyte cells. Each dot plot represents the concentration of one protein from ProcartaPlex™ Mouse Immune Monitoring Panel, 48plex. Results are shown as mean ± SD, (n = 6 for each time point). Statistics were determined using a Kruskal—Wallis Test with a Dunn Test post hoc, * *p* ≤ 0.05, ** *p* ≤ 0.01, *** *p* ≤ 0.001.
