# Supplementary material for: Cytokine Profile Analysis During Sialodacryoadenitis Virus and Mouse Hepatitis Virus JHM Strain Infection in Primary Mixed Microglia and Astrocyte Culture—Preliminary Research
Source: Cells. 2025 Apr 25;14(9):637. doi: 10.3390/cells14090637 (PMC12071255; doi:10.3390/cells14090637)
Supplement: Supplementary file 1 [file cells-14-00637-s001.zip › cells-3531817-supplementary.pdf]

Supplementary materials:

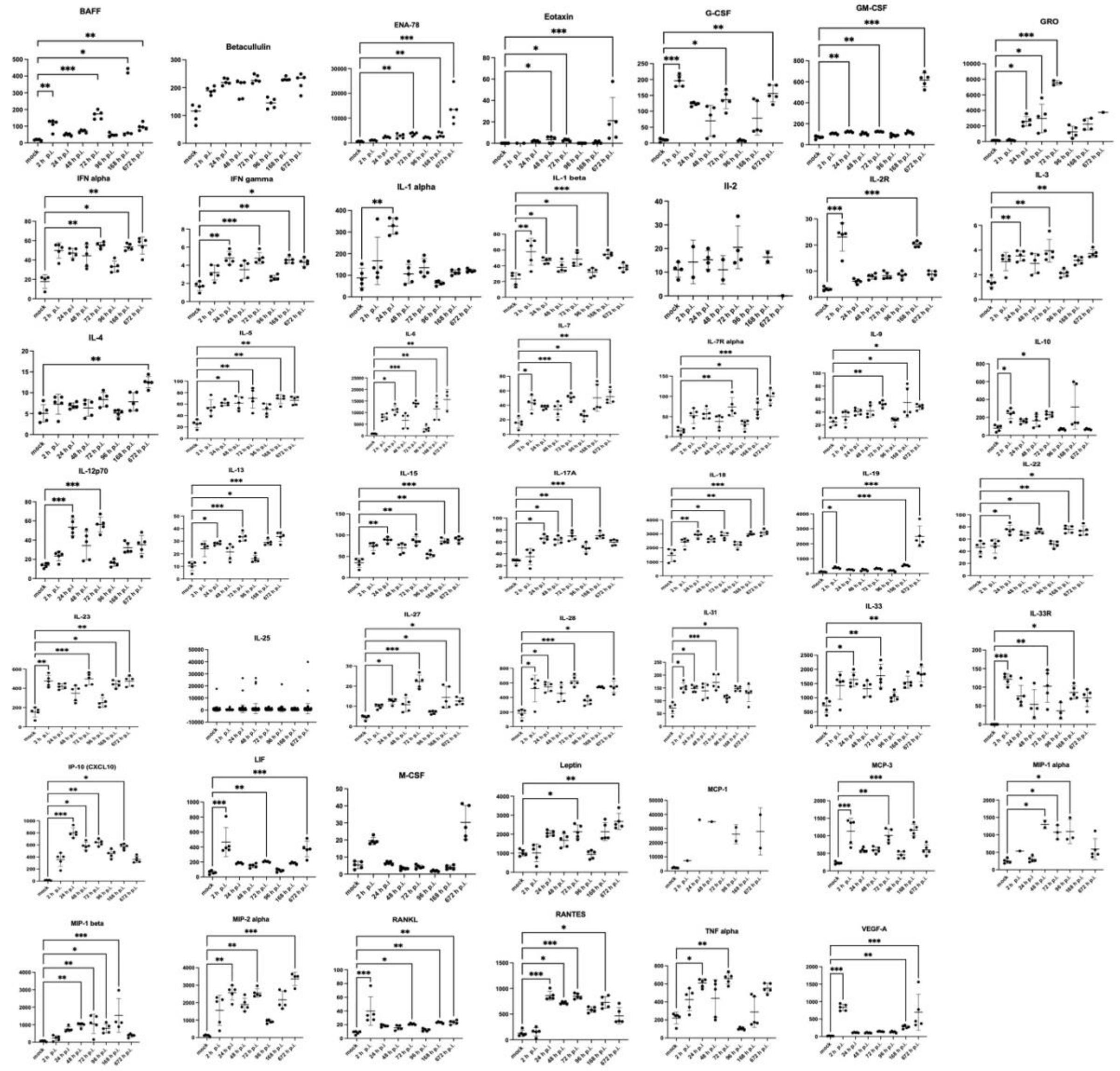

Figure S1. MHV-induced cytokine, chemokine, receptor and growth factor expression [pg/mL] in infected (2 – 672 hours) primary microglia and astrocyte cells. Each dot plot represents the concentration of one protein from ProcartaPlex™ Mouse Immune Monitoring Panel, 48plex. Results are shown as mean ± SD, (n = 6 for each time point). Statistics were determined using a Kruskal–Wallis Test with a Dunn Test post hoc, * p ≤ 0.05, ** p ≤ 0.01, *** p ≤ 0.001.

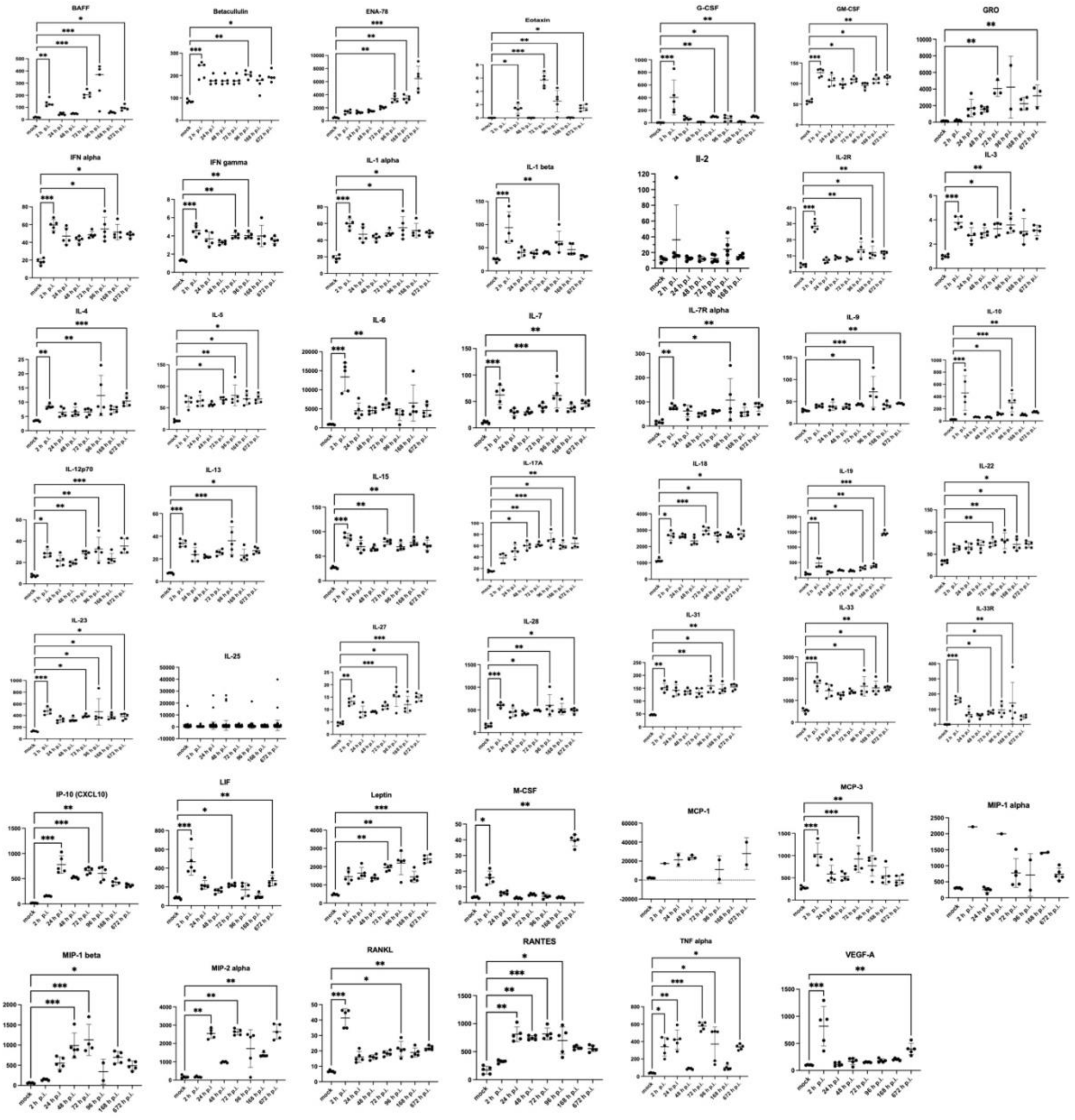

Figure S2. SDAV-induced cytokine, chemokine, receptor and growth factor expression [pg/mL] in infected (2 – 672 hours) primary microglia and astrocyte cells. Each dot plot represents the concentration of one protein from ProcartaPlex™ Mouse Immune Monitoring Panel, 48plex. Results are shown as mean ± SD, (n = 6 for each time point). Statistics were determined using a Kruskal–Wallis Test with a Dunn Test post hoc, * p ≤ 0.05, ** p ≤ 0.01, *** p ≤ 0.001.
